# Association between blood lead levels and hyperlipidemiais: Results from the NHANES (1999–2018)

**DOI:** 10.3389/fpubh.2022.981749

**Published:** 2022-09-09

**Authors:** Yangchang Zhang, Weiwei Liu, Wei Zhang, Rui Cheng, Andi Tan, Shisi Shen, Yang Xiong, Limei Zhao, Xun Lei

**Affiliations:** ^1^School of Public Health, Chongqing Medical University, Chongqing, China; ^2^Research Center for Medicine and Social Development, Chongqing Medical University, Chongqing, China; ^3^The Innovation Center for Social Risk Governance in Health, Chongqing Medical University, Chongqing, China; ^4^Research Center for Public Health Security, Chongqing Medical University, Chongqing, China; ^5^Chongqing College of Traditional Chinese Medicine, Chongqing, China; ^6^Research Center for Medicine and Social Development, Chongqing, China; ^7^Jiangxi Provincial Key Laboratory of Preventive Medicine, School of Public Health, Nanchang University, Nanchang, China; ^8^International Business School, Yunnan University of Finance and Economics, Kunming, China; ^9^The First School of Clinical Medicine, Chongqing Medical University, Chongqing, China; ^10^West China Hospital, Sichuan University, Chengdu, China; ^11^Chongqing Jiangjin Central Hospital, Chongqing, China

**Keywords:** blood, lead, hyperlipidemia, NHANES, environment pollution, adults

## Abstract

**Background:**

Research on the association between blood lead (Pb) and lipid biomarkers have yielded inconsistent results, and epidemiological studies on blood Pb levels and hyperlipidemia are scarce. The present study aimed to examine the association between blood Pb levels and hyperlipidemia in adults from the National Health and Nutrition Examination Survey (NHANES).

**Methods:**

A total of 43,196 participants in the NHANES from 1999 to 2018 were included in the final analysis. Hyperlipidemia was determined based on the National Cholesterol Education Program guidelines. Blood Pb levels were assessed using inductively-coupled plasma mass spectrometry. Weighted multivariable logistic regression analysis and subgroup analysis were conducted to determine the correlation between blood Pb levels and hyperlipidemia.

**Results:**

In the multivariable logistic regression model, high blood Pb levels were significantly associated with hyperlipidemia after adjusting for confounders (OR 1.41; 95%CI: 1.18–1.67). Furthermore, elevated blood Pb levels were associated with an increased risk of hyperlipidemia across the four quartile (Q) groups (Q1: OR 1.00; Q2: OR 1.16 [95%CI: 1.04–1.29]; Q3: OR 1.39 [95%CI: 1.21–1.59]; and Q4: OR 1.33 [95%CI: 1.15–1.54]; *P* for trend <0.05). Significant moderating effects were found in the subgroup analysis stratified by age, education, hypertension, and diabetes (*P* < 0.05). In sensitivity analysis, the ORs for hyperlipidemia across the quartiles of blood Pb levels were 1.00, 1.17 (95%CI: 1.05–1.30), 1.42 (95%CI: 1.24–1.62), and 1.38 (95%CI: 1.19–1.60) for Q1, Q2, Q3, and Q4, respectively (*P* for trend <0.001) after removing adults with arteriosclerotic cardiovascular disease, and the ORs were 1.00, 1.13 (95%CI: 1.01–1.25), 1.38 (95%CI: 1.21–1.56), and 1.32 (95%CI: 1.16–1.52) for Q1, Q2, Q3, and Q4, respectively (*P* for trend <0.001) after including pregnant women.

**Conclusion:**

The current study showed a positive association between blood lead levels and hyperlipidemia.

## Introduction

Hyperlipidemia has become an increasingly common concern in Europe and the US, as well as in developing countries. This condition includes various genetic and acquired disorders that are associated with high lipid levels, including cholesterol and triglycerides. From 2015 to 2018, approximately 12% of adults aged ≥20 years reported total cholesterol levels higher than 240 mg/dL, and nearly 17% reported high-density lipoprotein (HDL) cholesterol levels <40 mg/dL in the US (1). In the US, 28 million adults reported total cholesterol levels higher than 240 mg/dL (1). Hyperlipidemia increases the risk of cardiovascular disease and is a leading cause of stroke and death (2).

Lead (Pb) is a neurotoxic heavy metal that exists widely in the environment. The use of Pb has increased globally in the past decades, and the mining, smelting, and refining industries have led to substantial increases in environmental levels. Much of the Pb exposure mixtures come from a variety of industry products around the life space, including paint, ceramic, and piping materials (3). Pb is generally absorbed by the human body through ingestion and inhalation and accumulates in bone (4). It is estimated that adults could absorb 3–10% of water-soluble Pb at oral dose, whereas for children, the estimates may increase to 40–50% (4). Pregnancy was associated with higher absorption of Pb (5). Furthermore, mothers can transfer Pb to the fetus *via* the blood and to infants *via* breastfeeding (5). Pb toxicity damages the human central nervous system, and a previous report has shown that, in children, the amount of Pb adsorption is linked to lower intelligence and impaired motor function (4).

The association between Pb exposure and hyperlipidemia is inconsistent, and epidemiological studies on blood Pb levels and hyperlipidemia are rare. Evidence from rat experiments showed that exposure to Pb can cause oxidative stress, which induces alterations in lipid metabolism and gives rise to lipid disorders (6). This result indicates that exposure to Pb is associated with hyperlipidemia. Similarly, a longitudinal study in the US of 426 aging males showed that blood Pb levels were positively correlated with total cholesterol and HDL concentrations. Moreover, a national cross-sectional study in the US among adults found that exposure to Pb for long periods may significantly increase the risk of low-density lipoprotein, C-reactive protein, systolic blood pressure, and diastolic blood pressure (7). Moreover, in pregnant women, Pb exposure was significantly associated with increased non-HDL cholesterol and gamma-glutamyl transferase in the US (7). However, the artisans in Abeokuta, Nigeria were more likely to have high low-density lipoprotein levels than the control group, but there was no significant association with HDL cholesterol or triglycerides (8).

The purpose of this study was to examine the association between blood Pb levels and hyperlipidemia for 10 pooled cycles from the US NHANES to find potential evidence that could shed light on the contradictory association between lipid biomarkers and Pb exposure found in previous studies, and the limitation on hyperlipidemia associated with long-term Pb exposure.

## Methods

### Study design and population

The NHANES is an ongoing nationally representative health examination and nutritional status survey of adults and children in the US. The project consists of a series of cross-sectional waves that began in 1998 and mainly include five data components: demographic, dietary, examination, laboratory, and questionnaire. Interviews were carried out in each participant's home, and examination and laboratory testing were conducted in mobile examination centers. Detailed information regarding the continuous NHANES survey design can be found at http://www.cdc.gov/nchs/nhanes/index.htm. All study protocols were approved by the ethics review board of the National Center for Health Statistics, and written informed consent was obtained from all participants before any data collection.

**Figure 1:**
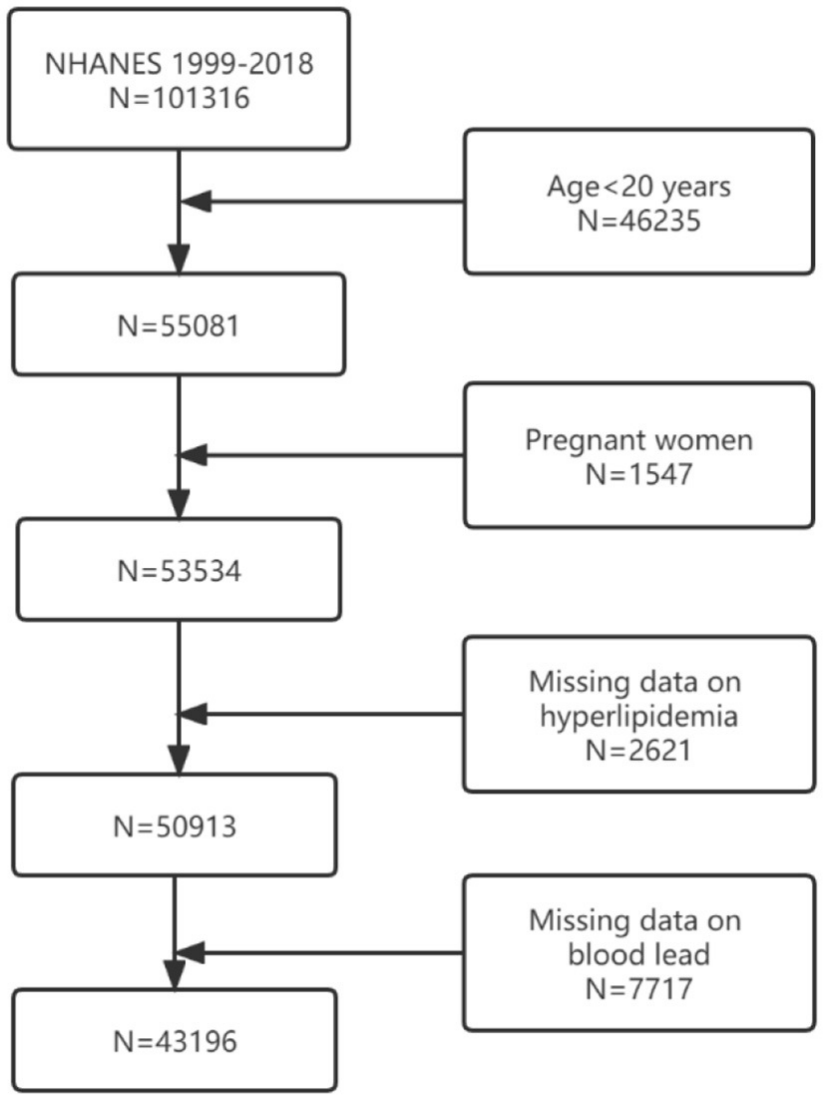
Sample selection process flow chart.

In the present study, we obtained data from 10 survey cycles (1999–2000, 2001–2002, 2003–2004, 2005–2006, 2007–2008, 2009–2010, 2011–2012, 2013–2014, 2015–2016, 2017–2018). In total, there were 101,316 participants in the pooled cycles. Participants with missing values for blood Pb levels and/or hyperlipidemia, or aged <20 years, or pregnant women, were excluded from the sample pool. The final sample for multiple cross-sectional analyses comprised 43,196 participants. The study flow chart is presented in Figure 1.

### Assessment of hyperlipidemia

We assessed the hyperlipidemia status based on the National Cholesterol Education Program (NCEP) in adults (Adult Treatment Panel III, ATP 3), which defined hyperlipidemia as triglycerides ≥ 150 mg/dL, total cholesterol ≥ 200 mg/dL, low-density lipoprotein ≥ 130 mg/dL, or HDL ≤ 40 mg/dL in males and ≤ 50 mg/dL in females (9). Alternatively, participants reporting use of cholesterol-lowering medications were also defined as having hyperlipidemia.

### Assessment of blood Pb concentration

Blood Pb levels reflect the systemic Pb load and Pb-exposed dose in the recent period. The method followed to measure blood Pb concentration was a modification of a previous approach. Whole blood samples were extracted, processed (10), stored, and shipped to the National Center for Environmental Health and Centers for Disease Control and Prevention for analysis. In the laboratory, specimens were diluted and then stored in −20°C conditions (11). Inductively-coupled plasma mass spectrometry was used to determine total whole blood Pb concentrations. This technique was based on quadruple ICP-MS technology (12). Blood Pb concentrations under the limit of detection (LOD) were replaced with the LOD divided by the square root of 2 (13). All blood Pb concentrations were determined for log2-transformation to an approximately normal distribution.

### Covariates

Based on previous research papers, potential confounding variables associated with blood Pb levels and hyperlipidemia were included in the final analyses (14, 15), including age, sex, race, marital status, education level, alcohol intake, smoking status, hypertension, ratio of family income to poverty, and diabetes mellitus. The participant age chosen for this study was that at the time of screening. Sex was dichotomized into male and female. Race was classified as Mexican American, non-Hispanic Black, non-Hispanic White, other Hispanic, and other race. Marital status was divided into living alone and married or living with a partner. Education level was defined as less than high school, high school, and above high school. The ratio of family income to poverty was the index used to estimate household socioeconomic status, which was graded as <1.5, 1.5–3.5, or >3.5 (16). Alcohol intake was classified as mild, moderate, and heavy. Heavy alcohol use was defined as ≥3 drinks per day for females or ≥4 drinks per day for males, or binge drinking on five or more days per month (12). Moderate alcohol use was defined as ≥2 drinks per day for females and ≥3 drinks per day for males, or binge drinking ≥2 days per month. Mild alcohol use was regarded as others (17). Smoking status was defined as never, former, and current (18). Never smokers were those who smoked <100 cigarettes in their lifetime; former smokers smoked >100 cigarettes in their lifetime but currently did not smoke at all; and current smokers smoked >100 cigarettes in their lifetime and currently smoked some days or every day. Hypertension was defined as an average blood pressure >140 mmHg systolic and/or 90 mmHg diastolic, report of hypertension diagnosed by a physician, or taking hypertension medicine. Physical activity level was calculated using the metabolic equivalent (MET, Per week) (19). The body mass index (BMI) was classified as underweight (<20 kg/m^2^), normal (≥20 to <25 kg/m^2^), overweight (≥25 to <30 kg/m^2^), and obese (≥30 kg/m^2^) (20). Diabetes mellitus was defined as reporting a diabetes diagnosis, glycohemoglobin HbA1c (%) > 6.5, or fasting glucose (mmol/L) ≥ 7.0, random blood glucose (mmol/L) ≥ 11.1, 2 h OGTT blood glucose (mmol/L) ≥ 11.1, or use of diabetes medication or insulin (21).

### Statistical analysis

Blood Pb concentrations were grouped into quartiles from lowest (first quartile, Q1) to highest (fourth quartile, Q4). The sample population was summarized by means and standard deviations for continuous variables and numbers and proportions for categorical variables. The difference among quartiles in blood Pb levels and sample characteristics was determined using one-way ANOVA analysis for normally distributed data and Chi-square test for categorical data. A 10-cycle sample weight (1999–2018) was utilized to account for over-sampling and non-responses. The primary sample unit and strata were defined to account for complex multistage probability sampling based on tutorials on the NHANES website. Three logistic regression models were conducted to estimate the associations of blood Pb concentration quartiles and hyperlipidemia. Crude model was no covariate; Model 1 was adjusted for age and sex; Model 2 was adjusted for age, sex, race, marital status, ratio of family income to poverty, educational level, alcohol intake, smoking status, BMI, hypertension, and diabetes mellitus. The effective strength of the associations was assessed by the odds ratio (OR) and 95% confidence intervals (CI).

Alternatively, some additional analyses were conducted. First, blood Pb concentrations were included in three logistic models as continuous variables, not quartiles. Second, interactive analyses were performed, stratified by other covariates. The *P* for interaction below 0.05 reveled significant interactive effects. Third, we examined the association between blood Pb concentrations and hyperlipidemia after excluding participants with arteriosclerotic cardiovascular disease (ASCVD is defined as having one or multiple cardiovascular disease, including coronary heart disease, angina, heart attack, and stroke). Fourth, we would keep pregnant group in the corresponding regression analysis. All statistical analyses were conducted using STATA software (Version 16.1, Stata Corporation) and R studio (Version 4.1.2). The significant threshold was defined as two-tailed *P* < 0.05.

## Results

There were 43,196 participants included in the final analysis across the quartiles of blood Pb levels, as shown in Table 1. Participants in the Q4 group tended to be older, female, high school education or above, non-Hispanic White, mild alcohol users, never smokers, overweight, with total MET >1,200 min/week, ratio of family income to poverty <1.5, no diabetes, and hypertension. There was a significant difference among the blood Pb quartiles in terms of age, sex, education, races, alcohol intake, smoking status, BMI, MET, ratio of family income to poverty, diabetes, hypertension and hyperlipidemia (*P* < 0.001).

**Table 1:** Characteristics of NHANES samples (1999–2018).

**Variables**		**Blood Pb levels quartiles**				***P*-value**
		**Q1**	**Q2**	**Q3**	**Q4**	
		***N* = 10,979**	***N* = 10,679**	***N* = 10,980**	***N* = 10,558**	
Age (years)		40.1 (15.8)	49.1 (17.3)	54.3 (17.0)	58.5 (16.8)	<0.001
Education	Above high school	7,919 (72.1%)	6,418 (60.1%)	5,838 (53.2%)	4,356 (41.3%)	<0.001
	High school	2,267 (20.6%)	3,077 (28.8%)	3,674 (33.5%)	4,107 (38.9%)	
	Less than high school	784 (7.1%)	1,171 (11.0%)	1,445 (13.2%)	2,074 (19.6%)	
	Missing	9 (0.1%)	13 (0.1%)	23 (0.2%)	21 (0.2%)	
Sex	Female	7,327 (66.7%)	5,901 (55.3%)	5,017 (45.7%)	3,516 (33.3%)	<0.001
	Male	3,652 (33.3%)	4,778 (44.7%)	5,963 (54.3%)	7,042 (66.7%)	
Races	Mexican American	1,855 (16.9%)	1,779 (16.7%)	1,922 (17.5%)	2,064 (19.5%)	<0.001
	Non-Hispanic Black	2,165 (19.7%)	2,077 (19.4%)	2,160 (19.7%)	2,459 (23.3%)	
	Non-hispanic white	4,712 (42.9%)	4,830 (45.2%)	5,228 (47.6%)	4,798 (45.4%)	
	Other hispanic	1,235 (11.2%)	950 (8.9%)	738 (6.7%)	528 (5.0%)	
	Other race—including multi-racial	1,012 (9.2%)	1,043 (9.8%)	932 (8.5%)	709 (6.7%)	
Alcohol intake	Heavy	1,815 (16.5%)	1,634 (15.3%)	1,655 (15.1%)	1,772 (16.8%)	<0.001
	mild	3,085 (28.1%)	3,239 (30.3%)	3,510 (32.0%)	3,131 (29.7%)	
	Moderate	1,925 (17.5%)	1,578 (14.8%)	1,511 (13.8%)	1,465 (13.9%)	
	Missing	4,154 (37.8%)	4,228 (39.6%)	4,304 (39.2%)	4,190 (39.7%)	
Smoking status	Former	1,884 (17.2%)	2,491 (23.3%)	3,115 (28.4%)	3,370 (31.9%)	<0.001
	Never	7,668 (69.8%)	6,134 (57.4%)	5,296 (48.2%)	4,033 (38.2%)	
	Now	1,424 (13.0%)	2,047 (19.2%)	2,560 (23.3%)	3,137 (29.7%)	
	Missing	3 (0.1%)	7 (0.1%)	9 (0.1%)	18 (0.2%)	
BMI (kg/m^2^)	0–20 (Underweight)	545 (5.0%)	431 (4.0%)	421 (3.8%)	561 (5.3%)	<0.001
	20–25 (normal)	2,567 (23.4%)	2,542 (23.8%)	2,761 (25.1%)	2,815 (26.7%)	
	25–30 (overweight)	3,019 (27.5%)	3,549 (33.2%)	3,873 (35.3%)	4,006 (37.9%)	
	30– (obese)	4,724 (43.0%)	4,001 (37.5%)	3,703 (33.7%)	2,896 (27.4%)	
	Missing	124 (1.1%)	156 (1.5%)	222 (2.1%)	280 (2.7%)	
Physical activity (MET, Per week)	<600	2,366 (21.6%)	2,615 (24.5%)	2,962 (27.0%)	2,770 (26.2%)	<0.001
	600–1,199	1,246 (11.3%)	1,275 (11.9%)	1,217 (11.1%)	1,080 (10.2%)	
	>1,200	4,833 (44.0%)	3,932 (36.8%)	3,630 (33.1%)	3,078 (29.2%)	
	Missing	2,534 (23.1%)	2,857 (26.8%)	3,171 (28.9%)	3,630 (34.4%)	
The ratio of family Income to poverty	0–1.5	3,473 (31.6%)	3,272 (30.6%)	3,388 (30.9%)	3,962 (37.5%)	<0.001
	1.5–3.5	3,356 (30.6%)	3,149 (29.5%)	3,297 (30.0%)	3,207 (30.4%)	
	3.5-	3,261 (29.7%)	3,349 (31.4%)	3,313 (30.2%)	2,358 (22.3%)	
	Missing	889 (8.1%)	909 (8.5%)	982 (8.9%)	1,031 (9.8%)	
Diabetes	No	8,615 (78.5%)	7,899 (74.0%)	8,195 (74.6%)	7,831 (74.2%)	<0.001
	Yes	2,364 (21.5%)	2,780 (26.0%)	2,785 (25.4%)	2,727 (25.8%)	
Hypertension	No	7,576 (69.0%)	6,163 (57.7%)	5,671 (51.6%)	4,832 (45.8%)	<0.001
	Yes	3,403 (31.0%)	4,516 (42.3%)	5,309 (48.4%)	5,726 (54.2%)	
Hyperlipidemia	No	3,890 (35.4%)	2,935 (27.5%)	2,647 (24.1%)	2,634 (24.9%)	<0.001
	Yes	7,089 (64.6%)	7,744 (72.5%)	8,333 (75.9%)	7,924 (75.1%)	

BMI, body mass index; MET, metabolic equivalent; Q, quartile.

All values are presented as proportion and number (%), or mean and SD.

High blood Pb levels were positively associated with hyperlipidemia in the null model, and the OR for hyperlipidemia across the quartiles of blood Pb levels was 1.00, 1.44 (95%CI: 1.34–1.54), 1.79 (95%CI: 1.65–1.95), and 1.85 (95%CI: 1.69–2.03) for Q1, Q2, Q3, and Q4, respectively. After adjusting for sex and age, the association was not significantly changed, and the OR of the blood Pb level quartiles was 1.00, 1.12 (95%CI: 1.04–1.20), 1.18 (95%CI: 1.08–1.29), and 1.08 (95%CI: 0.97–1.19) for Q1, Q2, Q3, and Q4, respectively. In the full mode, after further adjusting for race, education level, alcohol intake, smoking status, ratio of family income to poverty, diabetes, BMI, hypertension, and MET, the OR for hyperlipidemia across the blood Pb level quartiles was 1.00, 1.16 (95%CI: 1.04–1.29), 1.39 (95%CI: 1.21–1.59), and 1.33 (95%CI: 1.15–1.54) for Q1, Q2, Q3, and Q4, respectively (Table 2).

**Table 2:** Association between blood Pb and hyperlipidemia among US adults in NHANES 1999–2018.

	**Crude model**		**Model 1**		**Model 2**	
	**OR (95%CI)**	***P*-value**	**OR (95%CI)**	***P*-value**	**OR (95%CI)**	***P*-value**
Continuous	2.18 (1.95–2.44)	<0.001	1.07 (0.95–1.21)	0.236	1.41 (1.18–1.67)	<0.001
**Quartile**						
Q1	1		1		1	
Q2	1.44 (1.34–1.54)	<0.001	1.12 (1.04–1.20)	<0.002	1.16 (1.04–1.29)	0.008
Q3	1.79 (1.65–1.95)	<0.001	1.18 (1.08–1.29)	<0.001	1.39 (1.21–1.59)	<0.001
Q4	1.85 (1.69–2.03)	<0.001	1.08 (0.97–1.19)	0.153	1.33 (1.15–1.54)	<0.001
OR for trend	1.25 (1.21–1.29)		1.04(1.003–1.07)		1.12 (1.07–1.17)	
*P* for trend	<0.001		0.03		<0.001	

Crude model: no cofounder.

Model 1: adjusted for age and sex.

Model 2: adjusted for age, sex, race, education, alcohol intake, smoking status, body mass index, family poverty ratio, diabetes, hypertension, and physical activity.

OR, odds ratio; CI, confidence interval.

In the full model, the log-blood Pb concentrations were associated with a higher risk of hyperlipidemia (OR 1.41, 95%CI: 1.18–1.67). A significant linear association was found between the quartiles of blood Pb levels and hyperlipidemia (*P* for trend <0.001 and <0.05) (Table 2). No interaction of blood Pb levels and hyperlipidemia with the sex and BMI subgroups was identified (Table 3). Significant moderating effects were found in the subgroup analysis stratified by age, education, hypertension, and diabetes (*P* < 0.05), and it appeared stronger among participants aged below 60 years (OR 2.42, 95%CI: 2.03–2.89), among those with high school education or above (OR 2.57, 95%CI: 2.12–3.11), among those without diabetes (OR 2.59, 95%CI: 2.16–3.11) and hypertension (OR 2.35, 95%CI: 1.96–2.82) (Table 3).

**Table 3:** Subgroup analysis of association between blood Pb and hyperlipidemia among US adults in NHANES 1999–2018.

	**OR (95%CI)**	***P*-value**	***P* for interaction**
**Age (years)**			
<60	2.42 (2.03, 2.89)	<0.001	**0.03**
≥60	1.17 (0.75, 1.83)	0.48	
**BMI (kg/m** ^**2**^ **)**			0.07
BMI <20	2.49 (1.34, 4.61)	<0.01	
20 ≤ BMI <25	2.56 (1.93, 3.39)	<0.001	
25 ≤ BMI <30	2.28 (1.74, 2.98)	<0.001	
BMI≥ 30	1.81 (1.31, 2.48)	<0.001	
**Sex**			0.06
Male	2.31 (1.76, 3.02)	<0.001	
Female	2.15 (1.70, 2.72)	<0.001	
**Races**			0.18
Mexican American	1.83 (1.22, 2.75)	<0.01	
Non-Hispanic Black	1.65 (1.17, 2.30)	<0.01	
Non-Hispanic White	2.48 (2.00, 3.07)	<0.001	
Other Hispanic	1.78 (0.93, 3.41)	0.08	
Other Race	2.12 (1.22, 3.68)	<0.01	
**Education**			**0.001**
Above high school	2.57 (2.12, 3.11)	<0.001	
High school	1.54 (1.09, 2.18)	0.01	
Less than high school	1.49 (0.77, 2.87)	0.23	
**The ratio of family income to poverty**			0.30
0-1.5	2.11 (1.58, 2.81)	<0.001	
1.5-3.5	2.07 (1.56, 2.75)	<0.001	
3.5-	2.49 (1.90, 3.27)	<0.001	
**Physical activity (MET, per week)**			0.11
<600	1.77 (1.30, 2.39)	<0.001	
600-1199	2.11 (1.32, 3.35)	<0.01	
>1,200	2.54 (2.09, 3.10)	<0.001	
**Smoking status**			0.13
Formal	2.49 (1.74, 3.57)	<0.001	
Never	2.23 (1.76, 2.83)	<0.001	
Now	2.23 (1.76, 2.83)	<0.001	
**Alcohol intake**			0.16
Heavy	2.15 (1.56, 2.98)	<0.001	
Mild	1.98 (1.53, 2.58)	<0.001	
Moderate	2.80 (1.95, 4.02)	<0.001	
**Hypertension**			**<0.001**
Yes	1.37 (0.96, 1.95)	0.08	
No	2.59 (2.16, 3.11)	<0.001	
**Diabetes**			**<0.01**
Yes	1.32 (0.84, 2.10)	0.23	
No	2.35 (1.96, 2.82)	<0.001	

Adjusted for age, sex, race, education, alcohol intake, smoking status, body mass index, family poverty ratio, diabetes, hypertension, and physical activity except for subgroup variables. BMI, body mass index; CI, confidence interval; OR, odds ratio. The bold values mean significant *p* < 0.05.

An additional sensitively analysis was conducted in this study for which participants with arteriosclerotic cardiovascular disease were removed. In the full adjusted model, the OR for hyperlipidemia across the quartiles of blood Pb levels was 1.00, 1.17 (95%CI: 1.05–1.30), 1.42 (95%CI: 1.24–1.62), and 1.38 (95%CI: 1.19–1.60) for Q1, Q2, Q3, and Q4, respectively (*P* for trend <0.001) (Table 4). In the full analysis of including pregnant women, the OR for hyperlipidemia across the quartiles of blood Pb levels was 1.00, 1.13 (95%CI: 1.01–1.25), 1.38 (95%CI: 1.21–1.56), and 1.32 (95%CI: 1.16–1.52) for Q1, Q2, Q3, and Q4, respectively (*P* for trend <0.001) (Table 5).

**Table 4:** Association between blood Pb and hyperlipidemia excluding arteriosclerotic cardiovascular disease among US adults in NHANES 1999–2018.

	**Crude model**		**Model 1**		**Model 2**	
	**OR (95%CI)**	***P*-value**	**OR (95%CI)**	***P*–value**	**OR (95%CI)**	***P-*value**
Continuous	2.08 (1.85–2.35)	<0.001	1.11 (0.98–1.26)	0.095	1.49 (1.25–1.77)	<0.001
**Quartile**						
Q1	1		1		1	
Q2	1.41 (1.31–1.51)	<0.001	1.12 (1.05–1.21)	0.002	1.17 (1.05–1.30)	0.005
Q3	1.74 (1.60–1.90)	<0.001	1.20 (1.10–1.31)	<0.001	1.42 (1.24–1.62)	<0.001
Q4	1.77 (1.61–1.96)	<0.001	1.10 (0.99–1.23)	0.079	1.38 (1.19–1.60)	<0.001
OR for trend	1.23 (1.20–1.27)		1.05 (1.01–1.08)		1.13 (1.07–1.19)	
*P* for trend	<0.001		0.01		<0.001	

Crude model: no cofounder.

Model 1: adjusted for age and sex.

Model 2: adjusted for age, sex, races, education, alcohol intake, smoking status, body mass, index, the family of poverty ratio, diabetes, hypertension, and physical activity.

OR, odds ratio; CI, confidence interval.

**Table 5:** Association between blood Pb and hyperlipidemia including pregnant women among US adults in NHANES 1999–2018.

	**Crude model**		**Model 1**		**Model 2**	
	**OR (95%CI)**	***P* value**	**OR (95%CI)**	***P* value**	**OR (95%CI)**	***P* value**
Continuous	2.13 (1.91–2.37)	<0.001	1.05 (0.94–1.19)	0.351	1.40 (1.18–1.67)	<0.001
**Quartile**						
Q1	1		1		1	
Q2	1.39 (1.30–1.50)	<0.001	1.08 (1.002–1.15)	0.044	1.13 (1.01–1.25)	0.033
Q3	1.77 (1.64–1.92)	<0.001	1.16 (1.07–1.26)	0.001	1.38 (1.21–1.56)	<0.001
Q4	1.85 (1.69–2.02)	<0.001	1.07 (0.97–1.18)	0.186	1.32 (1.16–1.52)	<0.001
OR for trend	1.18 (1.16–1.20)		1.03 (1.001–1.07)		1.12 (1.07–1.17)	
*P* for trend	<0.001		0.043		<0.001	

Crude model: no cofounder.

Model 1: adjusted for age and sex.

Model 2: adjusted for age, sex, races, education, alcohol intake, smoking status, body mass, index, the family of poverty ratio, diabetes, hypertension, and physical activity.

OR, odds ratio; CI, confidence interval.

## Discussion

The independent association between blood Pb levels and hyperlipidemia was determined in this study. Our results show an increasingly significant association between blood Pb levels and hyperlipidemia examined after full adjustment. Additionally, subgroup analyses showed significant moderating effects for age, hypertension, and diabetes.

A linear association between blood Pb levels and hyperlipidemia was highlighted in this study. A positive association between blood Pb levels and cholesterol has been observed in previous studies. For example, a national cross-sectional study in the US showed that persistent occupational exposure to Pb could significantly increase the cholesterol levels of workers (7). Another large national cross-sectional study conducted in the US for 7 years found that Pb exposure could exacerbate oxidative stress in pregnant women and elevate cholesterol concentrations (22). The Korean National Environmental Health Survey, including 2,591 Korean adults, reported that blood Pb, blood mercury (Hg), and urine Hg levels were significantly correlated with dyslipidemia (23). The association between Framingham risk scores (FRS) and blood Pb levels was supported in the Korean cross-sectional study involving 1,929 participants, which showed that blood Pb levels were correlated with high systolic blood pressure, FRS, and total cholesterol (24) and with low HDL levels. Therefore, our findings support the previous evidence that blood Pb levels could increase the risk of hyperlipidemia.

The underlying mechanism of blood Pb on hyperlipidemia could be related to oxidative stress (25, 26). The levels of 8-epi-PGF2alpha, a biomarker of oxidative stress, were positively associated with hypercholesterolemia (26). Pb can lead to oxidative stress by binding the sulfhydryl protein group and depleting glutathione, then releasing active oxygen species (26). Oxidative stress can lead to the onset of hyperlipidemia by inducing an inflammatory reaction (27). This reaction was involved in the pancreatic β-cells' apoptotic process associated with insulin generation, which modified glycemic homeostasis, leading to insulin resistance (28). This process can result in endothelial dysfunction due to a decrease in vasodilator generation and increase in vasoconstrictor production (29). Experimental studies have also found that chronic exposure to Pb could impair glucose metabolism and disrupt glucose homeostasis *via* pancreatic β-cell damage (6, 30).

In the subgroup analysis stratified by age, participants aged <60 years showed a stronger association between blood Pb levels and hyperlipidemia than those aged ≥60 years. Our study design was based on a cross-sectional study that cannot determine the influence of Pb exposure on individual children and adults. Therefore, the effect of blood Pb levels on chronic disease stratified by age could be underestimated. Furthermore, the onset of hyperlipidemia is more common in the aging population due to alterations of body function, but the hyperlipidemia group among adults <60 years old might indicate a worse environment or unhealthy diet habits and lifestyles (31, 32). Meanwhile, the hyperlipidemia status of young adults moderated the risk of subsequent coronary heart disease with aging (33). Hence, age stratification magnified the direct effect of blood Pb on hyperlipidemia. No other research has completely revealed the underlying association between Pb blood levels and potential mediating biological processes in the development of coronary vascular disease and time-varying effects, such as age.

In terms of education stratification, participants above high school had a stronger association between blood Pb levels and hyperlipidemia than other people. The causal evidence using mendelian randomization had demonstrated that high education attainment was associated with lower cardiovascular disease and ischemic stroke (34). Low education was always linked with unhealthier life choices (such as irregular diet, high frequency of smoking, and alcoholism), thereby increasing the risk of getting cardiovascular disease (35–37). Additionally, Pb exposure was associated with poverty, older housing, African and Hispanic dominated communities (38). In our subgroup model, people would experience the adverse effects of Pb exposure to hyperlipidemia at all education stages. However, participants with high education had a stronger risk for hyperlipidemia while exposure to high Pb environment. This result indicated that the hazard of Pb exposure on hyperlipidemia may be further enlarged in the group without long-term experience of Pb exposure.

In the subgroup analysis stratified by diabetes and hypertension, participants with no diabetes or hypertension had a stronger association between blood Pb levels and hyperlipidemia. “Survival-Selection” mechanism demonstrated that frail aging people might be premature death due to multiple disease and worse medical service, and the leaving aging people had some stronger characteristics, such as genetic and behavior (39). Additionally, participants with chronic disease may pay more attention on medical knowledge, thus decreasing injury of the hazardous substances to human body. Meanwhile, the cross-section involving Chinese and American participants across all age-stage population demonstrated that the obesity-diabetes association significantly decreased with aging, indicating that the modification of diabetes might be closer in adults than older people (40). In a previous study, the positive association between Pb and blood pressure, not hypertension, was determined (41). Therefore, the group with continuous blood pressure increase was a potentially sensitive subpopulation in this relationship chain.

The current study has several strengths. First, a 10-cycle national cross-sectional sample was used in the analysis, which improves the statistical power. Thus, the weighted results could be interpreted as a national representative sample for American adults. Second, sensitivity analysis decreases the risk of false positives. In this study, we removed the participants with arteriosclerotic cardiovascular disease, because this group might always have hypercholesterolemia. Third, the blood Pb levels were divided into quartiles, which increases between-group heterogeneity and increases interpretability.

This study has several limitations. First, a causal association between blood Pb levels and hyperlipidemia cannot be verified due to the limitation of the cross-sectional NHANES data. Second, the adverse effect of chronic Pb exposure on the body cannot be completely explained in our study. Some participants could have had contact with environmental Pb for a long time prior to the surveys; therefore, the research of life courses can be reliable for estimating the long-term causal effects. Third, we only investigated the independent relationship between Pb blood levels and hyperlipidemia. However, the relationship between heavy metal exposure and disease is interactive. The complex network of multiple heavy metals, such as lead, mercury, and cadmium, could be further examined.

The large-scale national US survey was analyzed in this study. Our findings highlight the significant association between high blood Pb levels and hyperlipidemia after adjusting for confounders. However, the results cannot establish a causal association, and further studies are needed.

## Data availability statement

Publicly available datasets were analyzed in this study. This data can be obtained at: https://www.cdc.gov/nchs/nhanes/index.htm.

## Ethics statement

The studies involving human participants were reviewed and approved by the studies involving human participants were reviewed and approved by the National Center for Health Statistics Institutional Ethics Review Board, and all participants agreed on the survey and provided their written informed consent to participate in this study.

## Author contributions

YZ, WL, and XL contributed to the conception, analysis, interpretation of data, drafted the manuscript, and managed the final version for submission. WZ, RC, AT, SS, YX, and LZ contributed to analyzing and interpreting the data, commented on the report, and revised the manuscript. All authors have read and agreed on the published version of the manuscript. All authors contributed to the article and approved the submitted version.

## Funding

This work was supported by the National Natural Science Foundation of China (No. 72174033) and the Science and Technology Research Project of Chongqing Education Commission (No. KJQN202100432).

## Conflict of interest

The authors declare that the research was conducted in the absence of any commercial or financial relationships that could be construed as a potential conflict of interest.

## Publisher's note

All claims expressed in this article are solely those of the authors and do not necessarily represent those of their affiliated organizations, or those of the publisher, the editors and the reviewers. Any product that may be evaluated in this article, or claim that may be made by its manufacturer, is not guaranteed or endorsed by the publisher.

## Abbreviations

NHANES: National Health and Nutrition Examination Survey
Pb: lead
BMI: body mass index
OR: odds ratio
CI: confidence interval
MET: metabolic equivalent
ASCVD: arteriosclerotic cardiovascular disease.
